# Prevention Effect of Protopanaxadiol-Type Saponins Saponins and Protopanaxatriol-Type Saponins on Myelosuppression Mice Induced by Cyclophosphamide

**DOI:** 10.3389/fphar.2022.845034

**Published:** 2022-04-01

**Authors:** He Zhang, Lancao Zhang, Chunhui Yang, Yuyao Zhang, Jing Li, Xu Zhang, Jinjin Chen, Baotai Huang, Daqing Zhao, Xiangyan Li, Wei Zhang, Bin Qi

**Affiliations:** ^1^ Research Center of Traditional Chinese Medicine, The Affiliated Hospital to Changchun University of Chinese Medicine, Changchun, China; ^2^ Key Laboratory of Active Substances and Biological Mechanisms of Ginseng Efficacy, Ministry of Education, Jilin Provincial Key Laboratory of BioMacromolecules of Chinese Medicine, Jilin Ginseng Academy, Changchun University of Chinese Medicine, Changchun, China; ^3^ College of Pharmacy, Changchun University of Chinese Medicine, Changchun, China; ^4^ Office of Academic Research, Changchun University of Chinese Medicine, Changchun, China

**Keywords:** protopanaxadiol-type saponin, protopanaxatriol-type saponin, myelosuppression, cyclophosphamide, immunomodulatory effect

## Abstract

Ginsenosides from ginseng are used as a therapeutic agent for various diseases. They enhance the immunomodulatory effect in cyclophosphamide (CP)-treated tumor disease. The structural characteristics of steroidal saponins are mainly divided into protopanaxadiol-type saponin (PDS) and protopanaxatriol-type saponin (PTS). At present, few researchers have studied which kind of saponin plays a more important role, thus, we compared the prevention effect of PDS and PTS on myelosuppression mice induced by CP. The components and contents of saponin and monosaccharide were analyzed by using ultra high performance liquid chromatography-charged aerosol detector (UPLC-CAD) and reversed phase-high performance liquid chromatography (RP-HPLC), respectively. Thirty-two mice were randomly divided into four groups, including control, model (CP), CP+PDS, and CP+PTS. The mice were orally administered with PDS or PTS for 28 days and then injected with CP saline solution on 25, 26, 27, and 28 days at a dose of 50 mg × kg^−1^. After the end of modeling, the whole blood of mice from the ophthalmic venous plexus was collected to detect routine blood tests, inflammatory cytokines, and hematopoiesis-related cytokines. Cell cycle and the apoptosis of bone marrow in the right femur were detected. The spleen and thymus were used to calculate the organ index and histological examination, and splenocytes were used to detect the percentage of CD4^+^ and CD25^+^ T cells. In the saponins analysis, PDS mainly included the Rb1, Rc, Rb2, and Rd of protopanaxadiol-type ginsenosides (accounted for 91.64%), and PTS mainly included the Re, Rg1, and Rf of protopanaxatriol-type ginsenosides (accounted for 75.46%). The animal results showed that both PDS and PTS improved the most indicators of myelosuppression mice induced by CP, including increased weight, blood cell numbers, hematopoiesis-related cytokines, and inflammatory cytokines; promoted the cell cycle of bone marrow and inhibited the apoptosis of bone marrow; elevated the spleen and thymus indexes and CD4^+^ count of splenocytes. The prevention effect of PDS was better than PTS in some indicators, such as red blood cells, hemoglobin, interleukin (IL)-1β, IL-4, IL-10, tumor necrosis factor-α, CD4^+^, and thymus index. These results suggest both PDS and PTS can prevent myelosuppression of mice induced by CP. Meanwhile, PDS and its metabolite showed higher bioavailability and bioactivity compared with PTS.

## Graphical Abstract

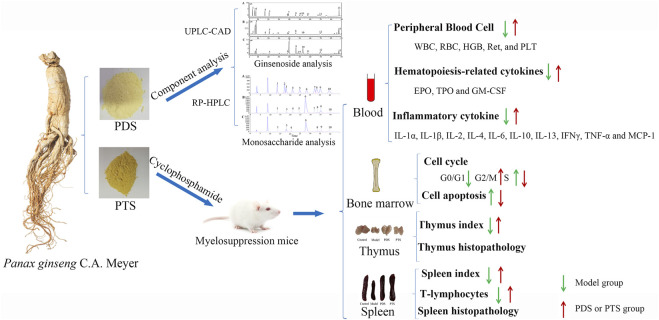

## 1 Introduction

Cyclophosphamide (CP) is a broad-spectrum chemotherapy drug used for various cancers. However, CP has severe side effects, such as nausea, vomiting, alopecia, anemia, bone marrow depression, and can cause injury of the spleen, thymus, heart, liver, and kidney (Bhat et al., 2018). Among these side-effects, myelosuppression is the most common, and it not only affects the chemotherapy regimen but also decreases life quality and increases economic burden (Afsar et al., 2012). Myelosuppression decreases peripheral blood cells, such as red blood cells (RBC), white blood cells (WBC), and platelets (PLT), causing anemia, thrombocytopenia, and neutropenia (Song et al., 2013). Therefore, strategies to effectively reduce the side reactions and protect the hematopoietic function of the bone marrow after chemotherapy will provide a better safeguard for patients.

In recent years, ginsenosides have received widespread attention. Ginsenosides are the important active components from the stem and leaf of *Panax ginseng* C.A. Meyer and have a broad range of beneficial effects including anti-tumor (Zhu et al., 2021), immunomodulatory (Liu et al., 2019), anti-inflammatory, anti-oxidant (Yao et al., 2019), anti-aging (Yu et al., 2020), anti-diabetes (Fan et al., 2019), anti-mutagenic(Varjas et al., 2009), neuroprotection (Zhang et al., 2017), and prevention and treatment of cardiovascular and cerebrovascular diseases (Lin et al., 2020). Saponins are composed of aglycones and monosaccharides, and common monosaccharides had glucose (Glc), arabinose (Ara), galactose (Gal), mannose (Man), galacturonic acid (GalA), rhamnose (Rha), ribose (Rib), glucuronic acid (GlcuA), xylitol (Xyl), and fucose (Fuc) in the ginsenosides (Yang et al., 2013). The structural characteristics of steroidal saponins are divided into protopanaxadiol-type saponin (PDS), protopanaxatriol-type saponin (PTS), oleanane-type saponin, and ocotillol-type saponin (Yang et al., 2013). Most ginsenosides in ginseng are PDS and PTS that belong to dammarane-type triterpenoids. The PDS mainly included Ra2, Rb1, Rb2, Rb3, Rc, Rd, and Rg3; and the PTS mainly included Re, Rf, Rg1, nR1, and Rh1 in the ginseng (Zhang and Shi, 2016). Many researchers have reported that total saponins or monomer saponin from ginseng regulate the body’s immune function, such as Re, Rg1, Rg3, CK, and Rk3 (Wei et al., 2012; Zhang et al., 2017; Han et al., 2019a; Han et al., 2019b; Fan et al., 2019; Liu et al., 2019). However, few studies have compared the therapeutic effect of PDS and PTS on myelosuppression mice.

This study analyzed the components and contents of ginsenosides in PDS and PTS to establish which were the main active ingredients. The effects of PDS and PTS on myelosuppression mice induced by CP were compared by evaluating their indicators, including body weight, peripheral blood cells, the hematopoiesis-related cytokines, inflammatory cytokines, cell cycle, and apoptosis of bone marrow, spleen and thymus index, and CD4^+^ and CD25^+^ T cell. Our results showed that PDS exhibited powerful prevention effects against CP-induced myelosuppression mice compared with PTS. Our results provide a new perspective for further research.

## 2 Materials and Methods

### 2.1 Chemicals

The reference substances of ginsenosides (nR1, Rg1, Re, pF11, Rf, Ra2, Rb1, Rc, Ro, Rb2, Rb3, Rd and 20(*R*)-Rg3) and monosaccharides (Glc, Ara, Gal, Man, GalA, Rha, Rib, ClcUA, Xyl and Fuc) were purchased from Shanghai Yuanye Biotechnology (Shanghai, China). The purity of these references was higher than 99.0% indicated by HPLC analysis. Cyclophosphamide (CP) was supplied by Sigma-Aldrich Company Ltd., (United States). Thrombopoietin (TPO), erythropoietin (EPO), and granulocyte-macrophage colony stimulating factor (GM-CSF) were purchased from Nanjing Jiancheng Bioengineering Institute (Nanjing, China). Quantibody®Array Glass Chip of IL-1α, IL-1β, IL-2, IL-4, IL-6, IL-10, IL-13, IFNγ, TNF-α and MCP-1 were purchased from RayBiotech Life, Inc. (United States). FITC-Annexin-V and Cell Cycle kits were purchased from BD Biosciences Pharmingen (United States). The blood cell analysis reagent kit was purchased from IDEXX Laboratories Inc (United States). Protopanaxadiol saponins (PDS) and protopanaxatriol saponins (PTS) were supplied by Professor Chen Yanping in the College of Chemistry, Jilin University; PDS and PTS were LC-MS grade. The voucher specimen of PDS (S20190011) and PTS (S20190012) were deposited in the authors’ lab in Changchun University of Chinese Medicine (Changchun, China).

### 2.2 Component Analysis of Protopanaxadiol-Type Ssaponin and Protopanaxatriol-Type Saponin

#### 2.2.1 Ginsenosides Content Analysis by UHPLC-CAD

The samples were analyzed on a Thermo Fisher Scientific UltiMate 3000 UHPLC + Focused × 2 dual gradient systems with a CORTECS UPLC Shield C18 column (2.1 mm × 100 mm, 1.6 µm). The column oven was maintained at 28°C, and the mobile phases included solvent A (water and 0.1% formic acid) and solvent B (acetonitrile). The following gradient was used: 0–7 min, 20% (B); 7–9 min, 20%–24% (B); 9–32 min, 24%–26% (B); 32–72 min, 26% (B); 72–80 min, 26%–35% (B); 80–86 min, 35%–50% (B); 86–92 min, 50%–60% (B); and 92–95 min, 60%–98% (B). The flow rate was 0.3 ml/min, and the injection volume was 3 µl for each sample. Data were carried out by using the Chromeleon 7.2 software (Thermo Fisher Scientific). The nitrogen pressure of CAD was adjusted to 60 psi and the response range was set to 100 pA. The other parameters, involving nebulization temperature of 40°C, filter constant of 3.6 s, and data collection rate of 5 Hz, were utilized. The quantitative assay experiments were performed using an external standard calibration method. A series of working solutions for 13 reference compounds were prepared from the stock solution by diluting with the appropriate volume of 70% aqueous methanol. The content of 13 ginsenosides were analyzed according to the standard curve (Xu et al., 2021).

#### 2.2.2 Monosaccharides Contents Analysis

The sample was hydrolyzed under vacuum in 4 mol/L trifluoroacetic acid for 2 h at 110°C. The monosaccharides that were converted to 1-Phenyl-3-methyl-5-pyrazolone (PMP) derivatives were analyzed by HPLC (Agilent 1200 series, Agilent Technologies, United States) with SHISEIDO C18 (4.6 mm × 250 mm, 5 µm). 0.1 mol/L KH_2_PO_4_ (pH6.8, eluent A) and acetonitrile (eluent B) were 82:18; flow rate was 1.0 ml/min. The detection wavelengths was 245 nm and the column temperature was 25°C. The solution was filtered through a syringe filter (0.22 µm) and the injection volume was 10 µl for each sample. A series of working solutions for 10 reference monosaccharides were prepared according to the derivative process above. The contents of 10 monosaccharides were analyzed according to the standard curve.

### 2.3 Animal Experiment

#### 2.3.1 Mouse Model

Male Kunming mice (weighing 20.0 ± 2.0 g) were purchased from Liaoning Changsheng Biotechnology Co., Ltd (Animal license No. SCXK (Liao)-2015-0001). The animals were housed under controlled temperature (25 ± 1°C), relative humidity (60 ± 5%), and a 12 h light/dark cycle with ad libitum access to food and water. This experiment was approved by the Bioethics Committee of Changchun University of Chinese Medicine and the Institutional Animal Care (Approval No. 2020247), which was conducted based on the guideline for the use of laboratory animals.

After 5 days of acclimatization, the mice were divided into four groups (*n* = 8 per group): normal control group (Control), CP treatment group (Model), CP+PDS (PDS, 0.10 g × kg^−1^), and CP+PTS (PTS, 0.10 g × kg^−1^). The mice were intragastrically (i.g.) given PTS and PDS at a dose of 0.10 g × kg^−1^, and the mice of the control and model groups were administered an equal volume of normal saline once a day for 28 consecutive days. The mice in the model, PDS, and PTS group were hypodermically injected with CP saline solution on 25, 26, 27, and 28 days at a dose of 50 mg × kg^−1^ (Li et al., 2014). After CP treatment, the experimental mice were fasted for 24 h.

#### 2.3.2 Peripheral Blood Cell

After fasting for 24 h, the whole blood of mice from the ophthalmic venous plexus was collected into the tube containing K2-EDTA. The blood cells were analyzed by the XT-2000i automated hematology analyzer (Sysmex Corporation, Japan).

#### 2.3.3 Inflammatory Cytokine Assay in Serum

Blood containing K2-EDTA was centrifuged at 4°C for 3,000 rpm × 10 min to obtain serum. Inflammatory cytokines were analyzed by the direct antigen-labeling technology (RayBio Biotin Label-based Antibody Array, RayBiotech) that included IL-1α, IL-1β, IL-2, IL-4, IL-6, IL-10, IL-13, IFNγ, TNF-α, and MCP-1.

#### 2.3.4 Determination of Hematopoiesis-Related Cytokines in Serum

The levels of erythropoietin (EPO), thrombopoietin (TPO), and granulocyte-macrophage colony stimulating factor (GM-CSF) of serum were measured by enzyme-linked immunosorbent assay (ELISA).

#### 2.3.5 Cell Cycle of Bone Marrow

The right femur was removed under aseptic conditions, and bone marrow cells were flushed using sterile phosphate-buffered saline (PBS) to form a single cell suspension, which was centrifuged after being rinsed twice with cold PBS. Then some of the cells were added to 2 ml of 70% cold ethanol, which were fixed at 4°C overnight. These cells were incubated with propidium iodide in the dark for 30 min and measured the cell cycle by flow cytometry.

#### 2.3.6 Cell Apoptosis of Bone Marrow

Other cells of bone marrow were centrifuged and fixed with 70% cold ethanol at 4°C overnight. They were washed twice with PBS to wash away the residual ethanol. The cell surface phosphatidylserine in apoptotic cells was quantitatively estimated using Annexin V-FITC and PI apoptosis detection reagent kit according to the manufacturer’s instructions by flow cytometry. A total of 10,000 events were acquired and at least three separate experiments were repeated for analyzing the cell apoptosis of bone marrow.

#### 2.3.7 Thymus and Spleen Index

After the animals were sacrificed, spleens and thymus were removed and weighed. Then, the thymus and spleen indices were calculated [organ weight (g)/body weight (g)].

#### 2.3.8 Splenic T-Lymphocyte Subpopulations Assay

Splenocytes were collected from mice splenic that were flushed using sterile PBS. The splenocyte suspension was adjusted to 1 × 10^6^ cells/mL and we measured the splenocyte lymphocyte subpopulations using flow cytometry. Splenocyte was labeled with FITC-conjugated anti-mouse CD4 and PE-conjugated anti-mouse CD25 (BioLegend). The labeled cells were washed twice and resuspended in staining buffer, and finally analyzed with DxFLEX (Backman, United States) and CellQuest software.

### 2.4 Statistical Analysis

All experiment data are shown as the mean ± standard deviations (SD). The significance of differences was analyzed by one-way ANOVA with Dunnett test and unpaired Student’s t-test using GraphPad Prism 8.0 software (GraphPad Inc., California, United States). *p* < 0.05 was considered as statistical significance.

## 3 Results

### 3.1 Component Analysis of Protopanaxadiol-Type Saponin and Protopanaxatriol-Type Saponin

**FIGURE 1:**
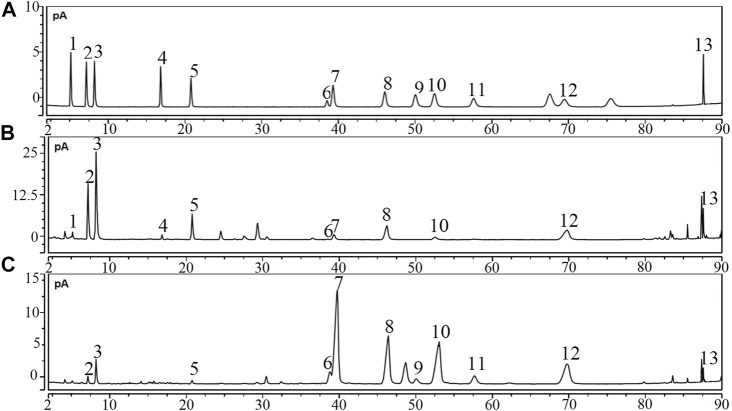
Chromatograms of PDS and PTS by UHPLC-CAD. **(A)** reference substances; **(B)** PDS; **(C)** PTS.

Saponins were the most important ingredient in PDS and PTS. They were detected by using the UPLC-CAD. Figure 1A shows the chromatograms of the 13 ginsenosides. According to the structural characteristics, ginsenosides were divided into protopanaxadiol-(PD), protopanaxatriol-(PT), oleanane- and ocotillol-type. In the 13 ginsenosides, Ra2, Rb1, Rc, Rb2, Rb3, Rd, and 20(*R*)-Rg3 belong to protopanaxadiol-type ginsenosides, and nR1, Rg1, Re, and Rf belong protopanaxatriol. pF11 and Ro belong to ocotillol- and oleanane-type ginsenosides, respectively. Figure 1B and Table 1 show the high-content protopanaxadiol-type ginsenosides in PDS, including Rb1, Rc, Rb2, and Rd. The content of protopanaxadiol-type ginsenosides accounted for 91.58% of the 13 ginsenosides. Ginsenoside nR1 and pF11 were not detected in PDS. Figure 1C and Table 1 show that the high-content protopanaxatriol-type ginsenosides in PTS included Re, Rg1, and Rf. The content of protopanaxatriol-type ginsenosides accounted for 77.10% of the 13 ginsenosides (Table 1). Ro and Rb3 were not detected in PTS. The above results show that the composition of ginsenosides had a significant difference between PDS and PTS.

**TABLE 1:** Ginsenoside Content of PDS and PTS as Determined by UHPLC (‾*x*± SD, *n* = 3).

NO.	Saponins	Type of saponins	PDS/mg × g^−1^	PTS/mg × g^−1^
1	Notoginsenoside R1 (nR1)	Protopanaxatriol	——	7.08 ± 0.51
2	Ginsenoside Rg1 (Rg1)	Protopanaxatriol	2.37 ± 0.20	129.19 ± 6.45
3	Ginsenoside Re (Re)	Protopanaxatriol	30.38 ± 1.08	334.74 ± 6.02
4	Pseudoginsenoside F11 (pF11)	Ocotillol	——	3.80 ± 0.15
5	Ginsenoside Rf (Rf)	Protopanaxatriol	19.33 ± 1.18	68.03 ± 3.02
6	Ginsenoside Ra2 (Ra2)	Protopanaxadiol	17.02 ± 0.76	2.12 ± 0.19
7	Ginsenoside Rb1 (Rb1)	Protopanaxadiol	225.78 ± 6.57	18.04 ± 0.31
8	Ginsenoside Rc (Rc)	Protopanaxadiol	151.09 ± 7.66	48.40 ± 2.22
9	Ginsenoside Ro (Ro)	Oleanane	12.16 ± 0.49	——
10	Ginsenoside Rb2 (Rb2)	Protopanaxadiol	165.52 ± 5.98	15.43 ± 0.32
11	Ginsenoside Rb3 (Rb3)	Protopanaxadiol	28.61 ± 0.62	——
12	Ginsenoside Rd (Rd)	Protopanaxadiol	107.63 ± 8.82	59.87 ± 3.30
13	20(*R*)- Ginsenoside Rg3 (20(*R*)-Rg3)	Protopanaxadiol	3.24 ± 0.19	12.36 ± 0.58
	Total content		768.45 ± 22.10	714.34 ± 7.60

Note: 1-nR1, 2-Rg1, 3-Re, 4-pF11, 5-Rf, 6-Ra2, 7-Rb1, 8-Rc, 9-Ro, 10-Rb2, 11-Rb3, 12-Rd, 13-20(*R*)-Rg3

**FIGURE 2:**
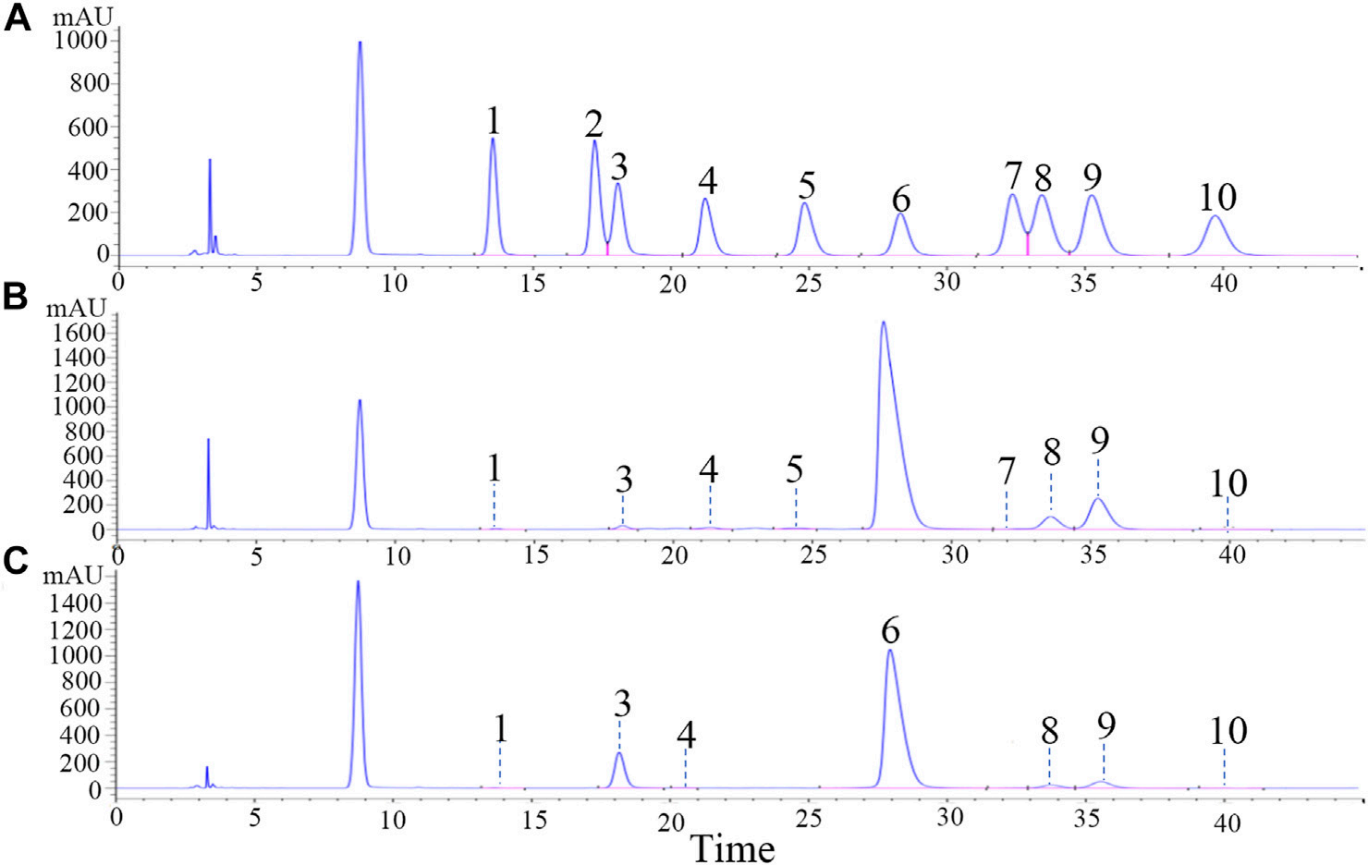
Monosaccharide composition analysis of PDS and PTS by HPLC. **(A)** reference substances; **(B)** PDS; **(C)** PTS.

Saponins were composed of aglycones and monosaccharides. Figure 2A shows chromatograms of the 10 reference monosaccharides. In Table 2, the total content of monosaccharides was very high in PDS and PTS at 751.58 mg/g and 803.32 mg/g, respectively. Among the monosaccharides, Glc content was highest, accounting for 87.49% and 84.76% of total monosaccharides in PDS and PTS, respectively. In Figure 2B and Table 2, besides Glc, the contents of Ara and Xyl were higher. Three monosaccharides accounted for 98.51% of total monosaccharides but the contents of Rha, GlcUA, GalA, Fuc, and Man were very low, and PDS did not contain Rib and Gal. In Figure 2C and Table 2, besides Glc, the content of Rha, Ara, and Xyl were higher in PTS, four monosaccharides accounted for 99.71% of total monosaccharides; the contents of Gal, GlcUA, Man, and Fuc were very low; and PDS did not contain Rib and GalA. The above results showed that the composition of monosaccharides had some differences between PDS and PTS.

**TABLE 2:** Monosaccharide content of PDS and PTS (‾*x*± SD, *n* = 3).

NO.	Monosaccharide	PDS/mg × g^−1^	PTS/mg × g^−1^
1	Mannose (Man)	0.32 ± 0.02	0.50 ± 0.04
2	Ribose (Rib)	——	——
3	Rhamnose (Rha)	6.18 ± 0.31	87.82 ± 2.24
4	Glucuronic acid (GlcUA)	5.28 ± 0.31	0.56 ± 0.56
5	Galacturonic acid (GalA)	2.73 ± 0.15	——
6	Glucose (Glc)	642.63 ± 18.48	580.90 ± 22.14
7	Galactose (Gal)	——	0.81 ± 0.01
8	Xylose (Xyl)	26.91 ± 1.34	10.35 ± 0.72
9	Arabinose (Ara)	66.71 ± 1.69	21.89 ± 1.52
10	Fucose (Fuc)	0.84 ± 0.05	0.48 ± 0.04
	Total content	751.58 ± 17.81	803.32 ± 17.98

Note: 1-Man, 2-Rib, 3-Rha, 4-GlcUA, 5-GalA, 6-Glc, 7-Gal, 8-Xyl, 9-Ara, 10-Fuc.

### 3.2 Effect of Protopanaxadiol-Type Saponin and Protopanaxatriol-Type Saponin on Body Weight and Organ Indexes in Myelosuppression Mice

**FIGURE 3:**
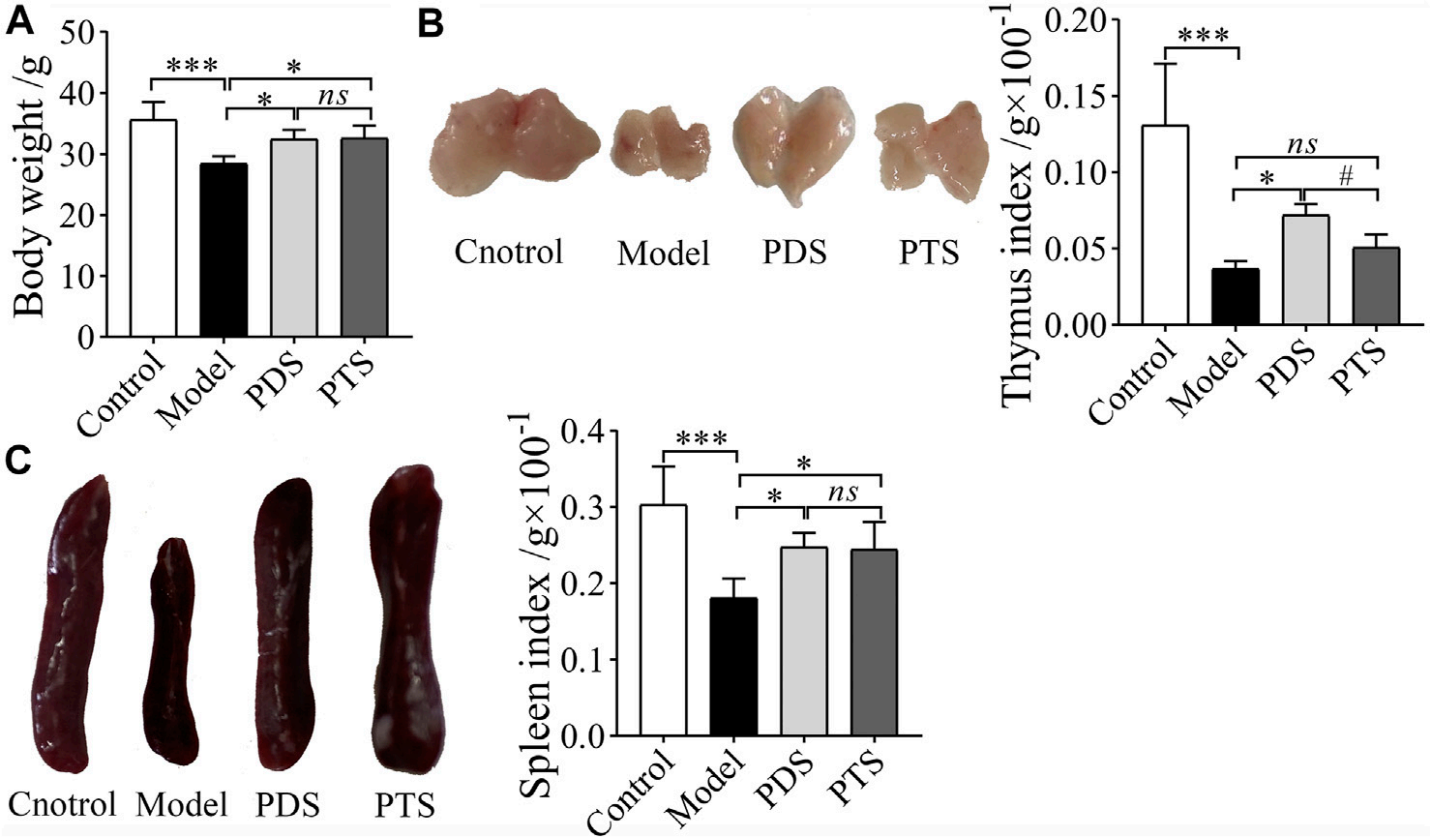
The effects of PDS and PTS on body weight and organ indexes in myelosuppression mice. **(A)** Body weight; **(B)** Thymus index; **(C)** Spleen index. The data are expressed as mean ± SD (*n* = 8), ^*^ *p* < 0.05 and ^***^ *p* < 0.001 compared with the model group; ^#^ *p* < 0.05 compared with PTS group, *ns* showed no significance between two groups.

The thymus and spleen are important immune organs; however, CP damages the thymus and spleen and weakens the immune function of the body (Sun et al., 2018). As shown in Figure 3, CP markedly reduced the body weight, thymus, and spleen indexes of mice (*p* < 0.001). The body weight, thymus, and spleen indexes of mice treated with PDS were higher than that of model mice (*p* < 0.05). Compared with the model group, PTS significantly prevented the reduction of body weight and spleen index in mice (*p* < 0.05). The thymus index of mice treated with PTS was higher than that of model mice, but there was no statistical difference (*p* > 0.05). Compared with the PTS group, the thymus index of mice was obviously higher in the PDS group (*p* < 0.05). According to the results above, PDS and PTS significantly prevented the reduction of body weight and atrophy of thymus and spleen in myelosuppression mice induced by CP, and the effect of PDS was superior to PTS in the thymus index.

### 3.3 Effect of Protopanaxadiol-Type Saponin and Protopanaxatriol-Type Saponin on Peripheral Blood Cells in Myelosuppression Mice

**FIGURE 4:**
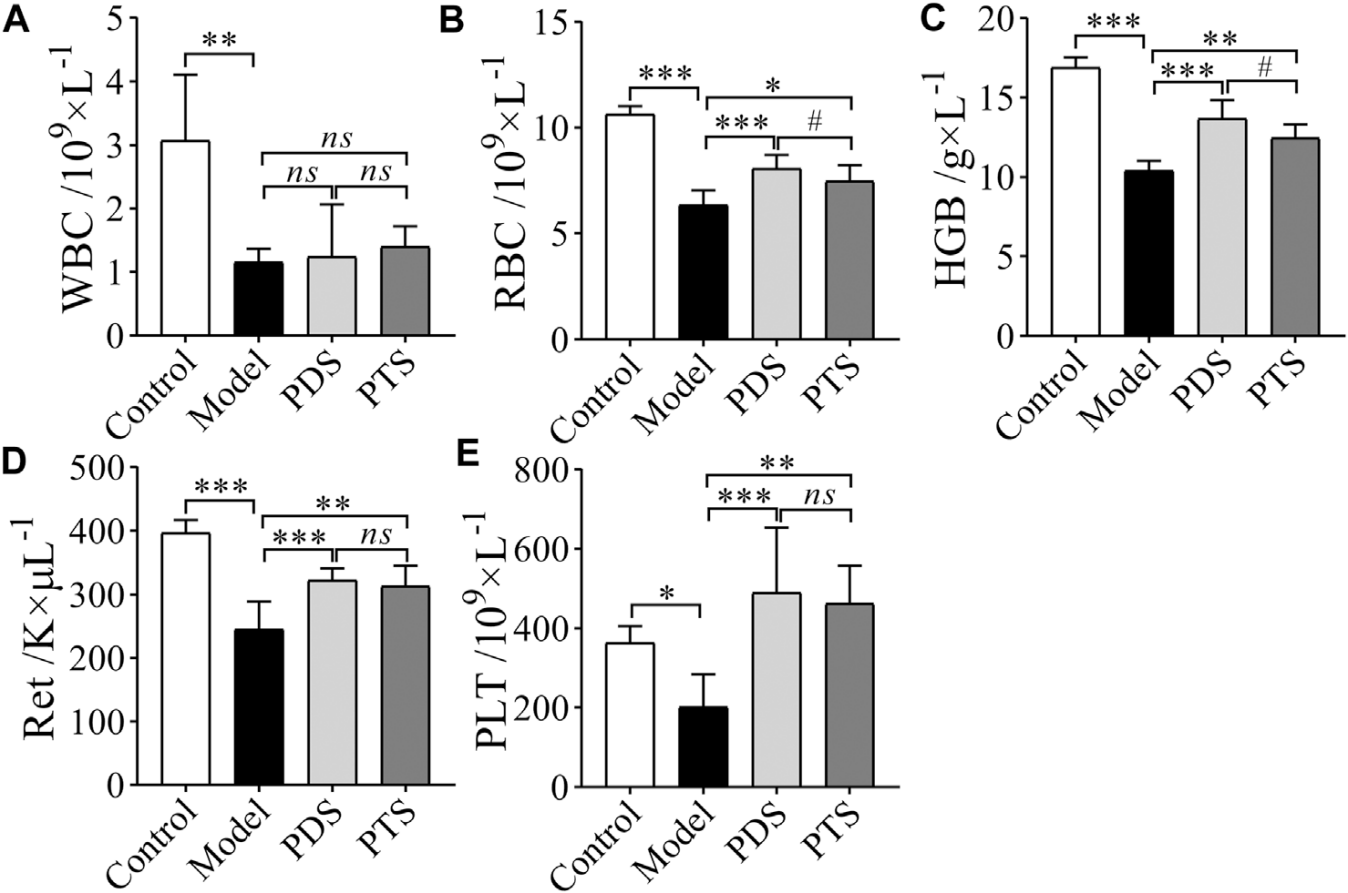
The effect of PDS and PTS on peripheral blood cells of myelosuppression mice. **(A)** White blood cell (WBC); **(B)** Red blood cell (RBC); **(C)** Hemoglobin (HGB); **(D)** Reticulocyte (Ret); **(E)** platelet (PLT). The data are expressed as mean ± SD (*n* = 8), ^*^ *p* < 0.05, ^**^ *p* < 0.01 and ^***^ *p* < 0.001 compared with the model group; ^#^ *p* < 0.05 compared with PTS group, *ns* showed no significance between the two groups.

As shown in Figure 4, the numbers of WBC, RBC, HGB, Ret, and PLT in mice after CP-induced were significantly decreased compared with the control group (*p* < 0.05, *p* < 0.01 or *p* < 0.001). The amounts of RBC, HGB, Ret, and PLT of the mice treated with PDS and PTS were higher than those of mice in the model group (*p* < 0.05, *p* < 0.01, or *p* < 0.001). However, both PDS and PTS did not inhibit the lessening of WBC of model mice (*p* > 0.05). The prevention effect of PDS on the RBC and the HGB of model mice was superior to PTS (*p* < 0.05). According to the results above, PDS and PTS prevented the reductions of RBC, HGB, Ret, and PLT in myelosuppression mice induced by CP, and PDS showed better amelioration than PTS in peripheral blood cells.

### 3.4 Effect of Protopanaxadiol-Type Saponin and Protopanaxatriol-Type Saponin on Erythropoietin, Thrombopoietin, and Granulocyte-Macrophage Colony Stimulating Factor in Myelosuppression Mice

**FIGURE 5:**
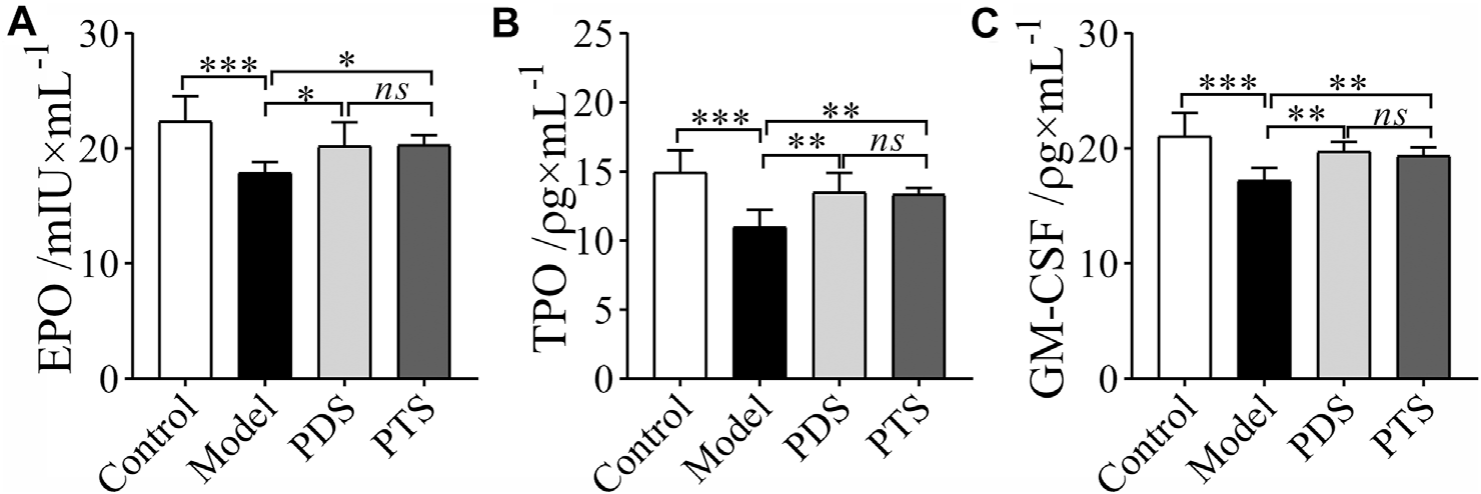
The effects of PDS and PTS on hematopoiesis-related cytokines in myelosuppression mice. **(A)** Erythropoietin (EPO); **(B)** Thrombopoietin (TPO); **(C)** Granulocyte-macrophage colony stimulating factor (GM-CSF). The data are expressed as mean ± SD (*n* = 5), ^*^ *p* < 0.05, ^**^ *p* < 0.01 and ^***^ *p* < 0.001 compared with the model group; *ns* showed no significance between the two groups.

CP had severe myelotoxicity, which caused hematopoietic dysfunction (Liu et al., 2015). Levels of EPO, TPO, and GM-CSF in serum were measured by ELISA kits. As shown in Figure 5, CP reduced the levels of EPO, TPO, and GM-CSF in mice (*p* < 0.001). In the PDS and PTS administration groups, the levels of EPO, TPO, and GM-CSF of mice were higher than those of model mice. Three indicators had no significance between PDS and PTS (*p* > 0.05). To sum up, PDS and PTS could prevent the reduction of hematopoiesis-related cytokines in myelosuppression mice.

### 3.5 Effect of Protopanaxadiol-Type Saponin and Protopanaxatriol-Type Saponin on Inflammatory Cytokines in Myelosuppression Mice

**FIGURE 6:**
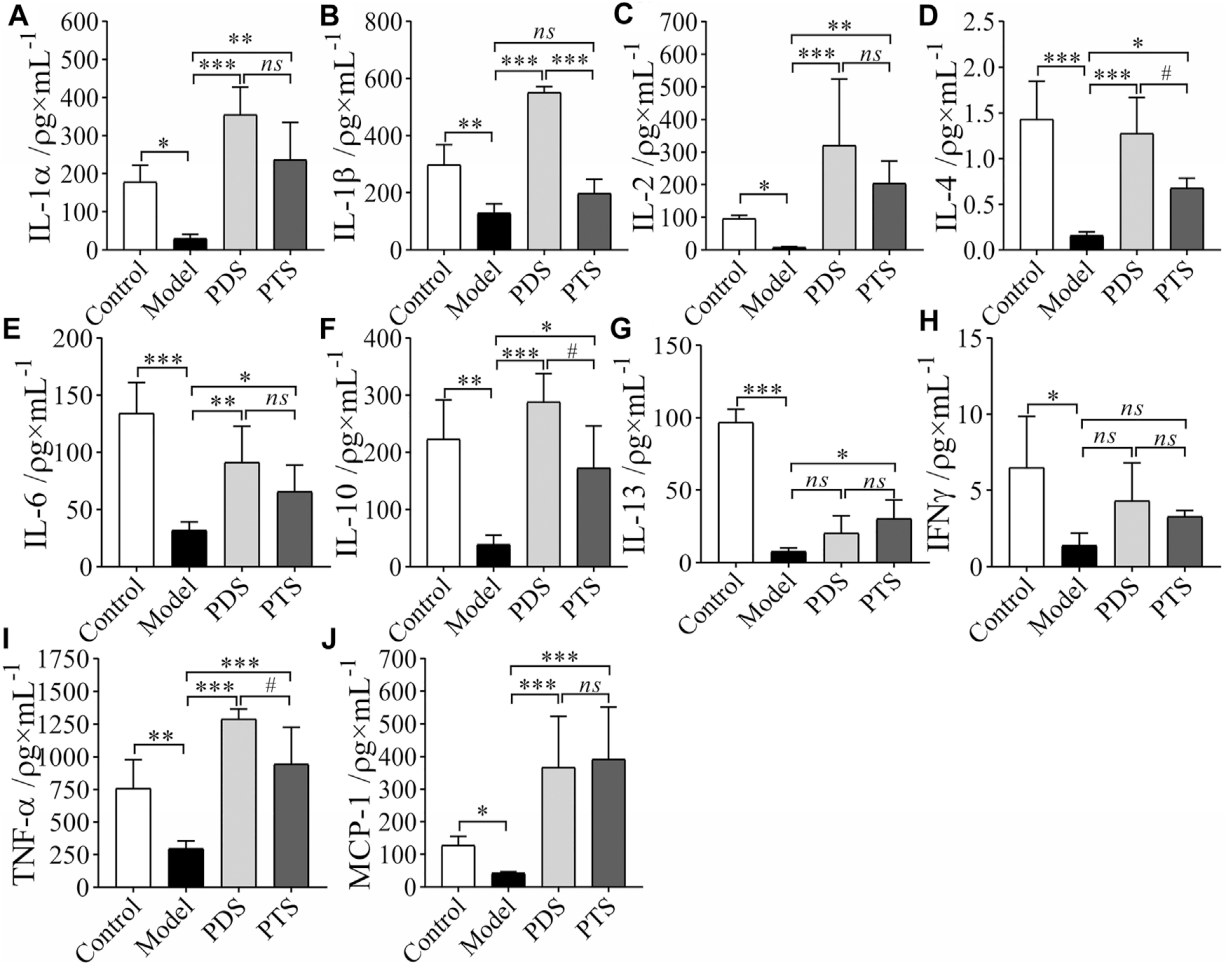
The effects of PDS and PTS on the levels of inflammatory cytokines in myelosuppression mice by using biotin-label-based antibody arrays. **(A)** IL-1α; **(B)** IL-1β; **(C)**, IL-2; **(D)**, IL-4; **(E)**, IL-6; **(F)**, IL-10; **(G)**, IL-13; **(H)**, IFNγ; **(I)**, TNF-α; **(J)**, MCP-1. The data are expressed as mean ± SD (*n* ≥ 4), ^*^ *p* < 0.05, ^**^ *p* < 0.01, and ^***^ *p* < 0.001 compared with the model group; ^#^ *p* < 0.05 compared with PTS group, *ns* showed no significance between the two groups.

Inflammatory cytokines are important factors in mediating the immune response (Bian et al., 1999). Therefore, to evaluate the balance of the body’s immune function, the key is to detect the secretion level of cytokine. In this article, inflammatory cytokines were detected by using biotin-label-based antibody arrays. In Figure 6, compared with the control group, levels of IL-1α, IL-1β, IL-2, IL-4, IL-6, IL-10, IL-13, IFNγ, TNF-α, and MCP-1 of mice in the model group significantly reduced (*p* < 0.05, *p* < 0.01 or *p* < 0.001). In the PDS group, IL-1α, IL-1β, IL-2, IL-4, IL-6, IL-10, TNF-α, and MCP-1 of mice were higher than those of mice in the model group (*p* < 0.05, *p* < 0.01 or *p* < 0.001). IL-13 and IFNγ increased but that had no significance compared with the model group (*p* > 0.05). PTS increased the release of IL-1α, IL-2, IL-4, IL-6, IL-10, IL-13, TNF-α, and MCP-1 of model mice (*p* < 0.05, *p* < 0.01 or *p* < 0.001), however, IL-1β and IFNγ had no significance compared with the model group (*p* > 0.05). IL-4, IL-10, and TNF-α had remarkable differences between PDS and PTS (*p* < 0.05). According to the results above, PDS and PTS prevented the reduction of most inflammatory cytokines in myelosuppression mice induced by CP, and the effect of PDS was better than that of PTS in the levels of IL-4, IL-10, and TNF-α.

### 3.6 Effect of Protopanaxadiol-Type Saponin and Protopanaxatriol-Type Saponin on Cell Cycle and the Cell Apoptosis of Bone Marrow in Myelosuppression Mice

**FIGURE 7:**
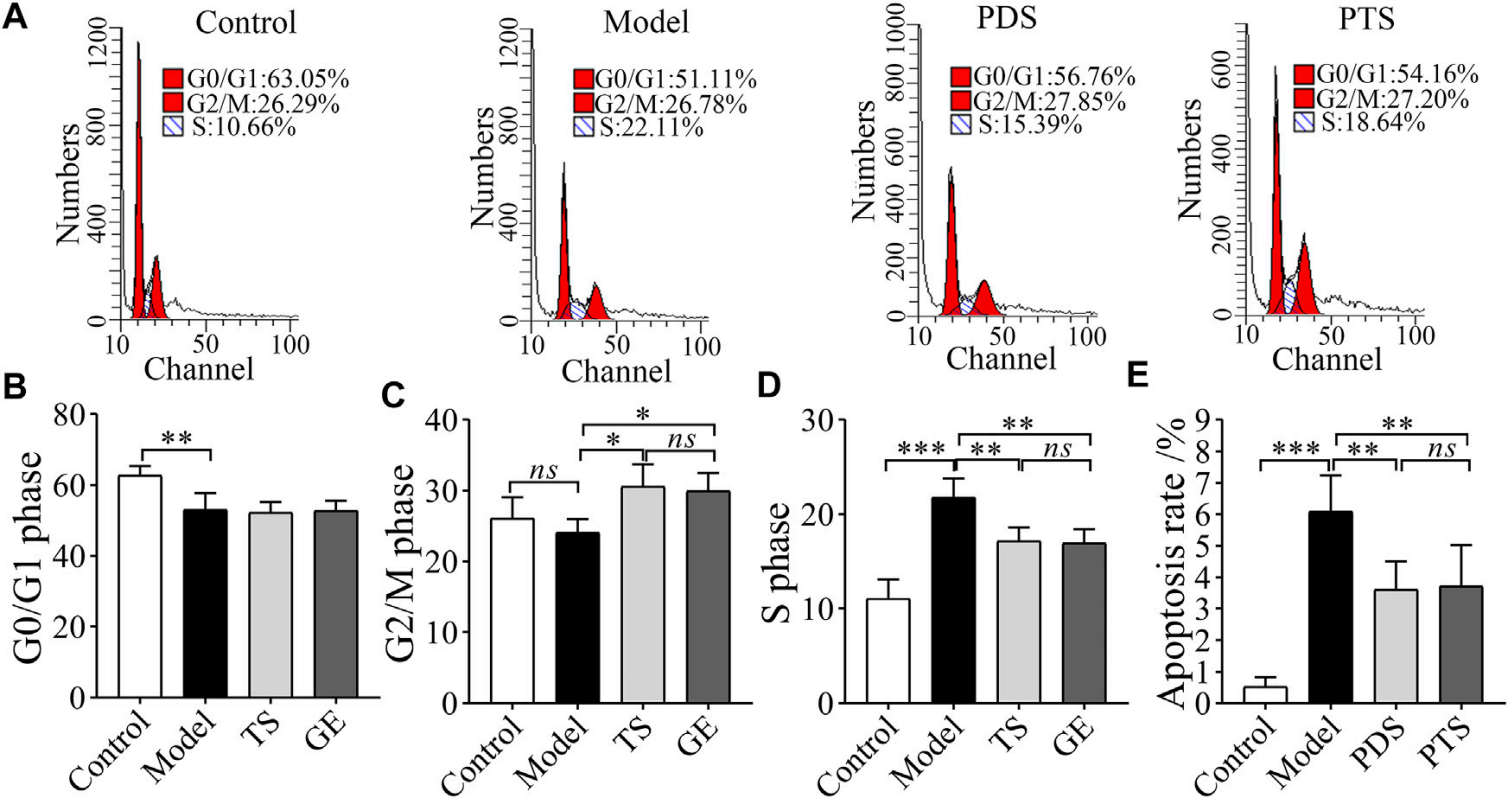
The effect of PDS and PTS on cell cycle and the apoptosis of bone marrow in myelosuppression mice. **(A)** Cell cycle scatter plot of bone marrow by flow cytometry. **(B)** analysis of G_0_/G_1_ phase; **(C)** analysis of G_2_/M phase; **(D)** analysis of S phase. **(E)** analysis of apoptosis rate. The data are expressed as mean ± SD (*n* = 3) *p < 0.05, ^**^ *p* < 0.01, and ^***^ *p* < 0.001 compared with the model group; *ns* showed no significance between the two groups.

CP damaged the bone marrow to lead to myelosuppression (Deng et al., 2018). As shown in Figure 7, CP reduced the percentage of G_0_/G_1_ phase (Figures 7A,B, *p* < 0.01) and increased the percentage of S phase (Figure 7C, *p* < 0.001). These results showed that CP induced an obvious S phase arrest. However, PDS and PTS significantly increased the G_2_/M phase (Figure 7C, *p* < 0.01) and decreased the S phase (Figure 7D, *p* < 0.01). In the cell apoptosis analysis of bone marrow (Figure 7E), CP increased the apoptosis rate of model mice (*p* < 0.001). The apoptosis rate of mice treated with PDS and PTS was less than that of model mice (*p* < 0.01). Collectively, PDS and PTS restored the normal cell cycle and inhibited the apoptosis rate of bone marrow in myelosuppression mice.

### 3.7 Effect of Protopanaxadiol-Type Saponin and Protopanaxatriol-Type Saponin on Histopathology of Thymus and Spleen in Myelosuppression Mice

**FIGURE 8:**
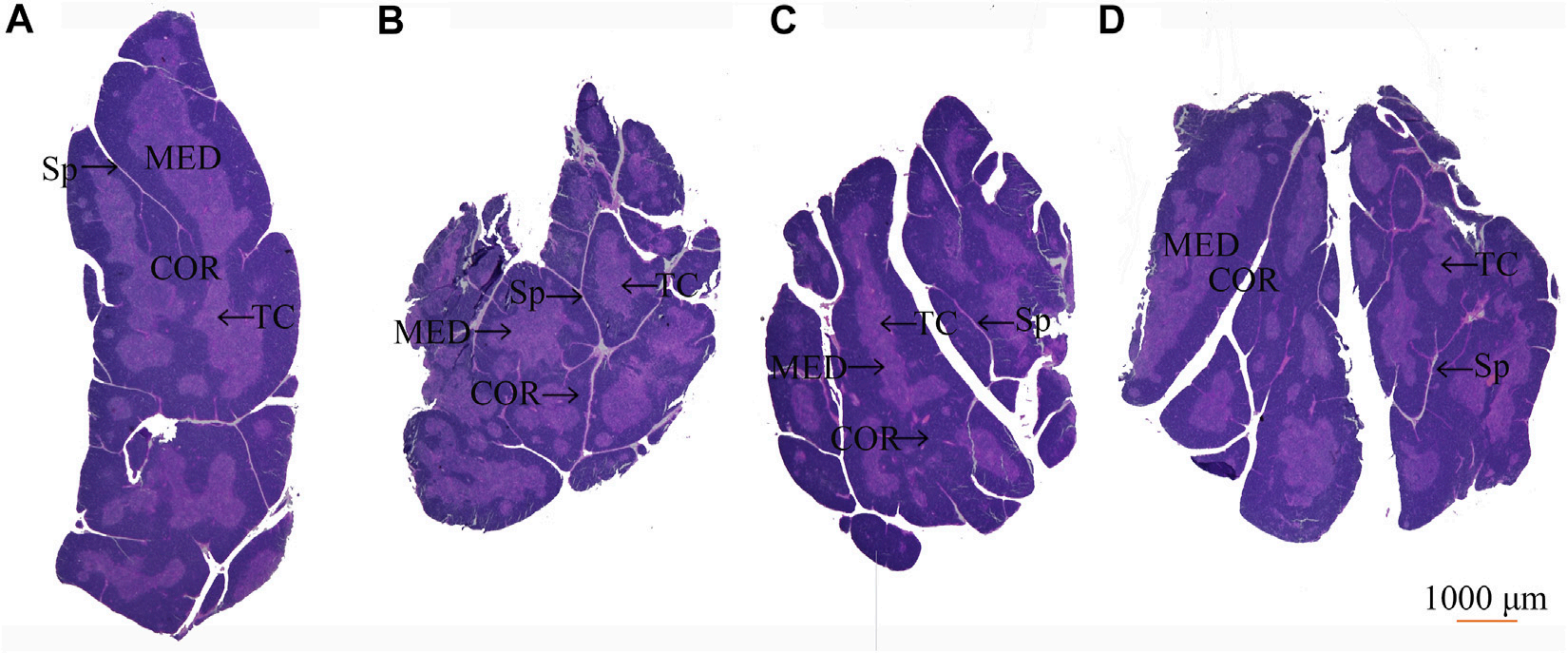
The effect of PDS and PTS on thymus histopathology in myelosuppression mice. **(A)** control group; **(B)** model group; **(C)** PDS group; **(D)** PTS group. (COR-cortex, MED-medulla, TC- thymic corpuscle, Sp-septum).

**FIGURE 9:**
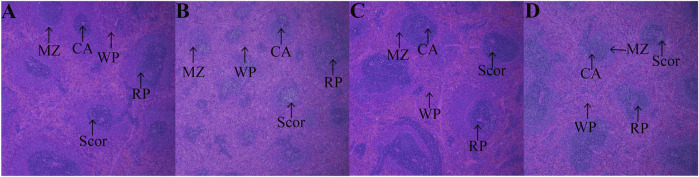
The effect of PDS and PTS on spleen histopathology in myelosuppression mice. **(A)** control group; **(B)** model group; **(C)** PDS group; **(D)** PTS group. (WP- white pulp, RP- red pulp, Scor-splenic corpuscle, CA-central artery, MZ-marginal zone).

After HE staining, compared with the control group (Figure 8A), the model group showed thymus gland atrophy, cortex (COR) thinning, medulla (MED), and septum (Sp) enlargement (Figure 8B). After treatment administration, the thymus lobules tended to be normal and showed clear separation of the COR and MED in the PDS and PTS groups. Dense thymocytes in the COR became deeper; round or oval thymic corpuscle (TC) could be seen obviously in the medullary substance (Figures 8C,D). In the histopathology of the spleen, compared with the control group (Figure 9A), Figure 9B shows that white pulp (WP) displayed weak staining, indicating attenuation and sparse lymphocyte. The splenic corpuscle (Scor) had slight atrophy and the central artery (CA) showed blurriness in the model group. Compared with the model group, the tint of WP became darker and well-distributed. Scor tended to be intact and the marginal zone (MZ) between WP and red pulp (RP) was clear in the PDS and PTS group (Figures 9C,D). These results showed that the number of lymphocytes was significantly increased, and the lymphocytes were arranged tightly. According to the results above, PDS and PTS prevented thymus and spleen damage from CP in myelosuppression mice.

### 3.8 Effect of Protopanaxadiol-Type Saponin and Protopanaxatriol-Type Saponin on CD4^+^ and CD25^+^ T-lymphocytes in Myelosuppression Mice

**FIGURE 10:**
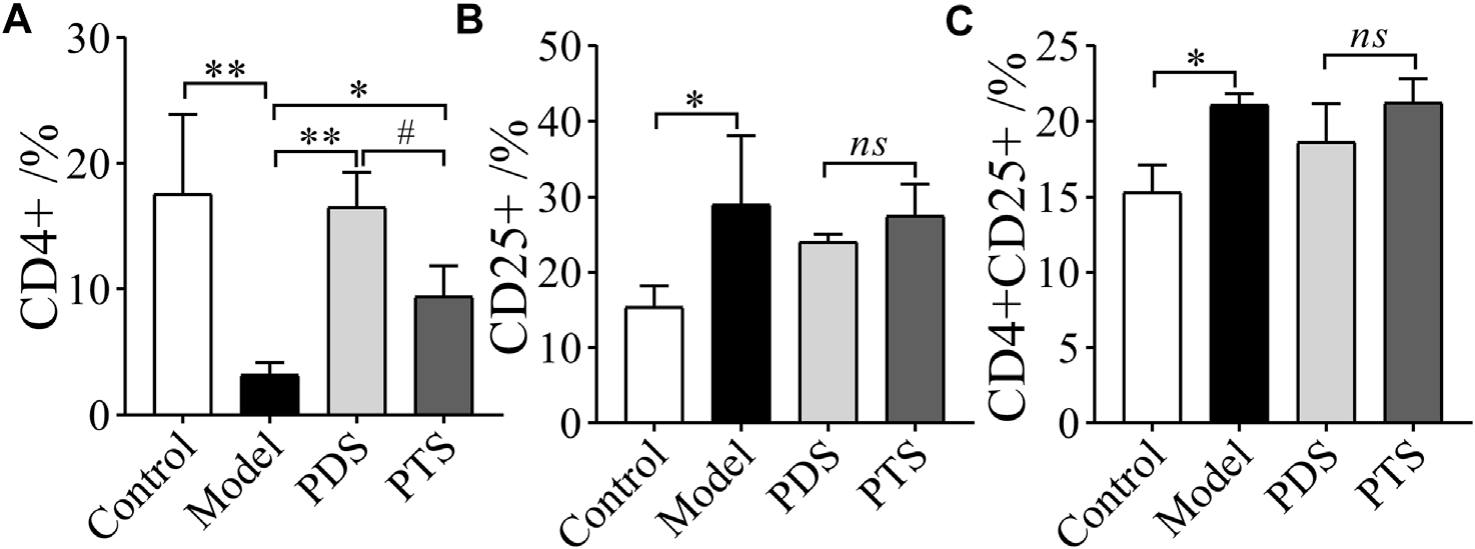
The effects of PDS and PTS on T-lymphocytes in myelosuppression mice. **(A)** CD4^+^; **(B)** CD25^+^; **(C)** CD4^+^CD25^+^. The data are expressed as mean ± SD (*n* = 3), ^*^ *p* < 0.05 and ^**^ *p* < 0.01 compared with the model group; ^#^ *p* < 0.05 compared with PTS group, *ns* showed no significance between the two groups.

T lymphocytes play a key role in suppressing immune responses, and a high dose of CP inhibits lymphocyte activity (Heylmann et al., 2013). As shown in Figure 10, CP significantly decreased the percentage of CD4^+^ and increases the percentages of CD25^+^ and CD4^+^CD25^+^ of model mice compared with the control group (*p* < 0.05 or *p* < 0.01). In the drug administration group, PDS and PTS significantly increased the percentage of CD4^+^ (Figure 10A, *p* < 0.05 or *p* < 0.01). The effect of PDS on CD4^+^ was stronger than that of PTS. However, PDS and PTS showed little improvement in CD25^+^ and CD4^+^CD25^+^ T-lymphocytes (Figures 10B,C, *p* > 0.05). PDS and PTS mainly improved the CD4^+^ T-lymphocytes of the spleen in myelosuppression mice induced by CP, and the effect of PDS was superior to PTS in the CD4^+^ T-lymphocytes.

## 4 Discussion

The immune system of the body consists of organs, cells, and cytokines. The important immune organs are bone marrow, thymus, and spleen. CP, a well-known chemotherapeutic drug, was used for tumor treatment; however, it causes adverse effects on the immune system to give rise to myelosuppression and immunosuppression, and even threatens human life (Bhat et al., 2018). In our study, CP inhibited the bone marrow of mice, including reduced peripheral blood cells (Figure 4), hematopoiesis-related cytokines (Figure 5) and inflammatory cytokines (Figure 6), increased apoptosis rate of bone marrow, and arrest S phase of the cell cycle (Figure 7), lessened the spleen and thymus (Figures 8, 9) and inhibited the activity of T-lymphocytes (Figure 10). These results were consistent with previous reports (Niu et al., 2020; Ying et al., 2020). In this study, we found that PDS and PTS remarkably improved these indicators of myelosuppression mice induced by CP, and the effect of PDS was superior to PTS in some indicators.

Bone marrow is responsible for immunity function by producing and maintaining the lifelong output of blood cells and immune cells (Schürch et al., 2021). CP caused myelosuppression, which resulted in cell cycle arrest and the apoptosis of bone marrow (Deng et al., 2018), and reduced the numbers of WBC, RBC, HGB, Ret, and PLT (Song et al., 2013). In this study, PDS and PTS prevented the reduction of RBC, HGB, Ret, and PLT in myelosuppression mice induced by CP (Figure 4). The improving effect of PDS on the RBC and HGB was better than that of PTS in model mice. However, both PDS and PTS did not inhibit the WBC reduction of model mice. CP badly affected the WBC of mice (Üstün et al., 2001), our results suggested that PDS and PTS could not prevent the WBC reduction of model rats in a short time. In the cell cycle and apoptosis, PDS and PTS significantly improved the S phase arrest and apoptosis rate of bone marrow in myelosuppression mice (Figure 7). Thus, the mice treated with PDS and PTS before modeling can prevent the myelosuppression of mice induced by CP.

In this article, hematopoiesis-related cytokines in serum including EPO, TPO, and GM-CSF were detected. EPO, an evolutionarily conserved hormone mainly produced in the kidney, promotes erythropoiesis (Peng et al., 2020). Wang et al. reported that EPO could directly affect the polarization of macrophages and tend to shift macrophages toward the M2 phenotype to exert anti-inflammatory function (Wang et al., 2017). TPO is the major regulator of platelet biogenesis by binding its receptor c-mpl, which could potentially reduce platelet and/or megakaryocyte apoptosis and therefore increase the platelet count (Lebois et al., 2016). GM-CSF is a hematopoietic growth factor responsible for the proliferation, differentiation, and maturation of cells of bone marrow (Fleetwood et al., 2005). Meanwhile, GM-CSF enhances the function of neutrophils, eosinophils, macrophages to improve anti-tumor and anti-infection (Lee et al., 2000; Wessendarp et al., 2021). CP damages the kidney or bone marrow to reduce the levels of EPO, TPO, and GM-CSF (Bhat et al., 2018; Sun et al., 2018). Our results showed that PDS and PTS prevented the reduction of EPO, TPO, and GM-CSF in myelosuppression mice to promote the production of blood cells and improve organ damage.

The thymus and spleen are important immune organs. Their indexes can reflect the nonspecific immunity of the body. CP damages the thymus and spleen to give rise to thymus and spleen atrophy and a reduction in their indexes (Sun et al., 2018). After treated administration, both PDS and PTS improved the morphology of the thymus and spleen and significantly increased the organ indexes of myelosuppression mice.

CD4^+^CD25^+^ regulatory T cell (Treg) mainly participate in suppressing immune responses (Heylmann et al., 2013). Treg prevents inflammation and autoimmune disorders by inhibiting the activity of T effector cells including CD4^+^ T helper cells (Th) and CD8^+^ cytotoxic T cells (Sakaguchi, 2000). Our results showed that PDS and PTS improved the CD4^+^ T cell of the spleen in myelosuppression mice. In several subtypes of Naïve CD4^+^ (antigen-inexperienced) T cell, the type 1 (Th1) and type 2 (Th2), T helper cells play important roles in the body against diseases (Elenkov and Chrousos, 2002). Th1 cells primarily secrete IL-1α/β, IL-2, IFNγ, and TNF-α during cell-mediated immune response; and Th2 cells secrete IL-4, IL-10, and IL-13 during a humoral-mediated immune response (Elenkov and Chrousos, 1999). IL-4 and GM-CSF potentiated IL-1β- and TNF-α-stimulated IL-8 and MCP-1 protein production in immune and non-immune cells (Bian et al., 1999). IL-1α/β, IL-2, IL-6, IFNγ, TFN-α, and MCP-1 belong to pro-inflammatory cytokines (Th1); IL-4, IL-10, and IL-13 belong to anti-inflammatory cytokines (Th2). Under the normal physiological conditions, Th1 and Th2 cells are in a dynamic equilibrium state to maintain normal cellular and humoral immune function. IL-1 is a family of polypeptide cytokines that has pleiotropic biological effects. IL-1 is involved in the host response to infection and inflammation (Dinarello et al., 2012). Dutcher et al. reported that IL-1 has the ability to both protect and restore the bone marrow from injury due to chemotherapeutic agents or radiation (Dutcher et al., 2014). IL-1α and IL-1β are key players in the innate immune system; IL-1α is implicated in the initiation/maintenance of the immune response to infection (Mehta et al., 2007); and IL-1β is produced by activated macrophages, T lymphocytes, and NK cells and is essential for the initiation of the immune response (Yoo et al., 2020). IL-2 is an important cytokine that stimulates the growth, proliferation, and differentiation of T and B cells, and mediates activation-induced cell death (Liao et al., 2011). IL-6 plays an important part in regulating these three arms of the immune response by limiting the Th1 response and promoting the Th2 and Th17 responses (Diehl et al., 2000). IFN-γ is one of the major immunoregulatory molecules inducing effective immune responses on macrophages (Schroder et al., 2006). IFN-γ activates antigen-presenting cells, upregulates transcription factor T-bet, and promotes the differentiation of Th1 cells (Liu et al., 2019). TNF-α, produced by almost all immune system cells and many other cell types, plays a pivotal role in the host defense system and can induce the expression of a number of other immunoregulatory and inflammatory mediators (Bradley, 2008). Monocyte chemoattractant protein-1 (MCP-1), a chemoattractant to T lymphocytes and monocytes, may play an important role in the pathogenesis of the idiopathic inflammatory myopathies (Liprandi et al., 1999). IL-4 acts on B cells to promote the proliferation and activation of B cells and facilitate the differentiation of immature T cells into Th2 cells, it is crucial for increasing the function of humoral immunity (Lafreniere et al., 2006). IL-10 is one of the major regulatory factors that modulates enhanced immune response. It can suppress pro-inflammatory cytokines, inhibit the proliferation of effector T cells and promote the maturation of Treg cells (Zhou et al., 2005; Dehghan et al., 2018). IL-13, a T cell-derived cytokine, acts on B cells and monocytes and inhibits inflammatory cytokine production (Wynn, 2003).In particular, the cytokines released from immune cells can stimulate innate immune responses, which are essential for immunomodulation. In this article, CP significantly reduced levels of IL-1α, IL-1β, IL-2, IL-4, IL-6, IL-10, IL-13, IFNγ, TNF-α, and MCP-1 of mice. The results showed that CP damaged the dynamic equilibrium, which inhibited the release of pro-inflammatory cytokines and the anti-inflammatory cytokines of mice, leading a reduction in the function of the immune system of the body. PDS and PTS obviously elevated most inflammatory cytokine levels of myelosuppression mice to normal physiological levels. These results suggest that both PDS and PTS stimulated the secretion of Th1 and Th2 cells to regulate the immune response and prevented the dynamic unbalance between the Th1 and Th2 cells in mice.

Ginsenosides from ginseng are used as a therapeutic agent for various diseases, which has been a focus in anti-tumor and immunomodulatory research in recent years. In this article, we mainly compared the prevention effect of PDS and PTS on myelosuppression mice induced by CP. According to the results, the prevention effect of PDS was better than PTS in some indicators, such as RBC, HGB, IL-1β, IL-4, IL-10, TNF-α, CD4^+^, and the thymus index. In the ginsenoside analysis, PDS mainly included the Rb1, Rc, Rb2, and Rd of protopanaxadiol-type ginsenosides (which accounted for 91.58%), and PTS mainly included the Re, Rg1, and Rf of protopanaxatriol-type ginsenosides (accounted for 77.10%). In the chemical structure, PDS and PTS belong to dammarane-type triterpenoids, however, the position and number of hydroxyl groups are different. PDS has C-3, C-12, and C-20 hydroxyl, and PTS has C-3, C-6, C-12, and C-20 hydroxyl. The site and composition of sugar substituents have some differences between PDS and PTS. The sugar substituent sites of PDS are in C-3 and C-20; however, PTS are in C-6 and C-20. In our results, the main monosaccharides of PDS were Glc, Ara, and Xyl, which did not contain Rib and Gal. The main monosaccharides of PDS were Glc, Rha, Ara, and Xyl, which did not contain Rib and GalA. The differences of PDS and PTS in chemical structure resulted in a great difference in bioavailability, in which PTS were quickly absorbed and eliminated compared with PDS in the body (Leung and Wong, 2010; Kong et al., 2013). Yu *et al* reported that the potential accumulation of PDS was higher than that of PTS in 6 months of study of the kinetic profiles of SHENMAI injection in dogs (Yu et al., 2017). Hu *et al* reported that area under the curve (AUC) of Rb1 was higher than that of Rg1 in rats after intravenous injection (iv) 10 mg/kg Rg1 or Rb1 (Han et al., 2007; Hu et al., 2011); meanwhile, Rb1 showed a higher plasma protein binding rate (80.11%–89.69%) compared with Rg1 (6.56%–12.74%) (Xu et al., 2017). Some studies have shown that Rb1 transformed into gypenoside XVII, ginsenoside Rd, ginsenoside F2, compound K, and PPD by intestinal bacteria (Quan et al., 2011). Kim *et al* reported that the main metabolites of Rg1 were Rd and CK in rats, and the latter were distributed throughout the organs (Lee et al., 2005; Kim et al., 2014). The metabolites of saponins are easily absorbed in rat plasma, which has higher bioavailability and bioactivity than the prototype compounds of ginsenosides (Lee et al., 2005; Dong et al., 2017). Jin *et al* reported that the T_max_ values of Rd, Rh2, CK, and PPD of red ginseng extract were increased in human plasma; however, Re and Rg1 (PPT-type ginsenosides) were not detected, because Re and Rg1 were metabolized to PPT by intestinal microbiota before the absorption occurs (Park et al., 2017), and the biotransformation rate of PTS could be faster than that of PDS (Jin et al., 2019). Rb1, Rc, Rb2, and Rd belong to protopanaxadiol-type ginsenosides (PDS), their pharmacokinetic parameters in the body are similar (He et al., 2015); and Re, Rg1, and Rf belong to protopanaxatriol-type (PTS). Their metabolism and absorption in the body are similar to Rg1 (Wu et al., 2015). In summary, because PDS could be well absorbed, and has a higher plasma protein binding rate and bioavailability; meanwhile, PDS could transform Rd and CK with high bioavailability and bioactivity in the body. However, PTS was absorbed and eliminated quickly in the body. The prevention effect of PDS was better than that of PTS.

In conclusion, PDS and PTS all improved the myelosuppression mice by CP, which could increase the body weight, blood cell number (RBC, HGB, Ret, and PLT), and the hematopoiesis-related cytokines (EPO, TPO, and GM-CSF), increase the levels of inflammatory cytokines to prevent the dynamic unbalance between the Th1 and Th2 cells, promote the cell cycle of bone marrow, inhibit the cell apoptosis of bone marrow, elevate the spleen and thymus index and CD4^+^ count of splenocytes. In some indicators, PDS showed the prevention effect compared with PTS, because PDS and its metabolite showed high bioavailability and bioactivity.

## Data Availability

The original contributions presented in the study are included in the article/Supplementary Material, further inquiries can be directed to the corresponding authors.
